# Network functional connectivity and anterior cingulate cortex gamma-aminobutyric acid in antipsychotic medication-naïve first-episode psychosis patients

**DOI:** 10.1017/S0033291726104358

**Published:** 2026-06-09

**Authors:** Genelle Dano Samson, Jose Omar Maximo, Eric Nelson, Seyedeh Nasim Adnani, Adil Bashir, Adrienne Carol Lahti

**Affiliations:** 1 University of Florida, USA; 2 The University of Alabama at Birmingham, USA; 3 Department of Electrical and Computer Engineering, Auburn University, USA; 4 Department of Psychiatry and Behavioral Neurobiology, Heersink School of Medicine, The University of Alabama at Birmingham, Birmingham, USA

**Keywords:** psychosis, first episode psychosis (FEP), schizophrenia, gamma-aminobutyric acid (GABA), anterior cingulate cortex, functional connectivity, resting state functional magnetic resonance imaging (rs-fMRI), magnetic resonance spectroscopy (MRS), antipsychotic medication-naive

## Abstract

**Background:**

Functional connectivity (FC) is consistently altered in patients with schizophrenia. The brain’s primary inhibitory neurotransmitter, gamma-aminobutyric acid (GABA), and its relationship to FC in psychosis spectrum disorders are under-investigated. The anterior cingulate cortex (ACC) has been implicated in many cognitive functions impaired in psychosis. We hypothesize that the relationships between ACC GABA and FC in key brain networks will be altered in first-episode psychosis (FEP) patients as compared to healthy controls (HC).

**Methods:**

We used magnetic resonance spectroscopy (MRS) with a MEGA-PRESS sequence to quantify ACC GABA levels in 67 antipsychotic medication-naïve FEP patients and 110 HC. Resting state functional magnetic resonance imaging (fMRI) was used to assess positive and negative FC within the default mode (DMN), salience (SN), dorsal attention (DAN), and executive control (ECN) networks. We used linear regressions to test GABA–FC relationships in each network between groups.

**Results:**

FEP patients had significantly lower GABA levels compared to HC. We also found several clusters in the ECN, DAN, and DMN where FC differed between groups. Ultimately, we found significant GABA–FC group interactions in two ECN clusters and one SN cluster, where GABA and FC were positively correlated in HC but negatively correlated in FEP.

**Conclusions:**

Our data add to the growing literature supporting GABA’s significant role in psychosis spectrum disorders, especially as it relates to FC in key brain networks. Our findings call for further investigation of the mechanisms underlying altered neurometabolic activity and connectivity in psychosis spectrum disorders.

## Introduction

Functional connectivity (FC), the temporal correlation between spatially distinct neurophysiological events as measured by functional magnetic resonance imaging (fMRI), has consistently identified brain networks wherein spatially distinct regions are reliably activated together, both at rest and for specific cognitive processes (Fox et al., 2005). Studies have implicated aberrant connectivity in schizophrenia (Demirlek et al., 2024; Manoliu et al., 2014; Menon, Palaniyappan, & Supekar, 2023; Williamson & Allman, 2012) (SZ), whose core symptoms include disordered thinking, altered perception, abnormal emotion, motivation, and social interaction. Examining FC in the networks underlying these functions will help elucidate the mechanisms that bring about the symptoms of psychosis spectrum disorders.

During a baseline state, or rest, neural activity increases in the default mode network (DMN), which monitors the internal mental landscape and processes internally directed cognition (Fingelkurts & Fingelkurts, 2011; Greicius, Krasnow, Reiss, & Menon, 2003; Gusnard, Raichle, & Raichle, 2001). During stimulus-driven cognitive processing, the DMN is deactivated, while the dorsal attention network (DAN), which controls and maintains attention (Kamran et al., 2014), and the executive control network (ECN), which is responsible for working memory and processing externally directed cognition (Seeley et al., 2007), are activated. Integral to the balance and mediation of these networks is the salience network (SN), which is responsible for the attentional capture of biologically and cognitively relevant stimuli, and the appropriate allocation of neuronal processing resources (Menon, 2011; Menon & Uddin, 2010; Menon, Palaniyappan, & Supekar, 2023). In psychosis spectrum disorders (PSD), cognitive function is altered, implying dysfunction in these networks (Menon, Palaniyappan, & Supekar, 2023). Positive symptoms, such as hallucinations and delusions, along with cognitive disorganization, are prominent clinical features of PSD (Kahn et al., 2015). These features may be indications of failure to properly integrate stimuli, misattribution of salience to irrelevant stimuli, and therefore aberrancies in these functional networks, especially in the SN (Drukker et al., 2024; Howes et al., 2020). Notably, the hubs of the SN, the insula and dorsal anterior cingulate cortex (dACC), have consistently been found to be abnormal in PSD, in both structure and function (Carter, MacDonald, Ross, & Stenger, 2001; Lahti et al., 2006; Liloia et al., 2021; Wang et al., 2024; Williamson & Allman, 2012).

Gamma-aminobutyric acid (GABA) is the primary inhibitory neurotransmitter in the central nervous system, and its role in PSD is not well understood, nor is its relationship to FC. Studies have suggested that GABA concentration levels are related to multiple aspects of intrinsic connectivity, including seed-based connectivity profiles, spatial composition, and inter-network temporal relationships (K. Wang et al., 2020), though the neurochemical mechanisms underlying network connectivity are not well established (Reddy-Thootkur, Kraguljac, & Lahti, 2022). Neurotransmission of glutamate, the primary excitatory neurotransmitter in the brain, drives the majority of total energy consumption in the brain, and is modulated by GABAergic interneurons (Petroff, 2002). Hypofunction of N-methyl-D-aspartate receptors (NMDAR) is hypothesized to be a key mechanism in the pathophysiology of PSD (Kraguljac et al., 2021; Olney & Farber, 1995), and NMDAR activity on GABAergic interneurons has been implicated in disrupting the excitation/inhibition balance, leading to the neurobiological alterations shown in PSD (Coyle, 2012; Moghaddam, Adams, Verma, & Daly, 1997; Moghaddam & Javitt, 2012).

Inhibitory neurons which use GABA are integral to regulating neuronal activation and allocating energy within networks (Bitanihirwe et al., 2009), and so the role of GABA in network connectivity is of particular interest in studying psychosis, which has been consistently linked with abnormal connectivity (Demirlek et al., 2024; Goodkind et al., 2015). Postmortem studies suggest GABA abnormalities by decreased expression of the mRNA integral to GABA synthesis (Gonzalez-Burgos, Hashimoto, & Lewis, 2010; Lewis, Hashimoto, & Volk, 2005; Thompson, Weickert, Wyatt, & Webster, 2009; Volk et al., 2000) and transport/reuptake (de Jonge, Vinkers, Hulshoff Pol, & Marsman, 2017; Guidotti et al., 2000), as well as increased density of postsynaptic GABA receptors (Benes, Vincent, Marie, & Khan, 1996; Volk et al., 2002). Animal studies further support the involvement of GABA in PSD symptomatology (Amitai, Kuczenski, Behrens, & Markou, 2012; Fujikawa, Yamada, & Jinno, 2021; Wozniak et al., 2018). Little is known about GABA pathology at the early stages of illness, especially prior to treatment (Reddy-Thootkur, Kraguljac, & Lahti, 2022). There is an increasing number of studies using magnetic resonance spectroscopy (MRS) to investigate GABA in PSD, but heterogeneity is considerable—imaging technology (magnet strength) and techniques (macromolecule suppression) vary, and status of patients when investigated (stage of illness, antipsychotic medication exposure, acuity of illness) varies even more widely (Reddy-Thootkur, Kraguljac, & Lahti, 2022). A number of MRS studies investigating GABAergic neurotransmission in PSD have supported abnormalities in ACC GABA levels, but findings differed—patient GABA concentrations were found to be higher (Ongur et al., 2010), lower (Bojesen et al., 2020; Bojesen et al., 2021), or not different (Tayoshi et al., 2010), as compared to healthy controls (HC). In a group of medication-naïve first-episode psychosis (FEP) patients, we found reduced levels of ACC GABA in comparison to matched HC, thus replicating Bojesen’s finding in a similar group of medication-naïve FEP patients (Bojesen et al., 2020; Samson et al., 2025). When comparing patients with FEP to HC, levels of glutamate and GABA in the ACC have been differentially associated with blood-oxygen-level-dependent (BOLD) response in the DMN (Overbeek et al., 2019), as well as with resting state FC in regions across the cortex (Overbeek et al., 2021) and in higher-order functional brain networks (Maximo et al., 2024).

The relationship between GABA levels within the ACC, a region implicated in several complex cognitive functions frequently impaired in PSD, and FC within and between key brain networks may play a pivotal role in the pathophysiology of PSD. We aim to investigate these relationships using MRS to measure ACC GABA, along with fMRI to measure resting state connectivity in the DMN, DAN, ECN, and SN. We hypothesize that in a group of medication-naïve FEP, we will replicate findings of decreased ACC GABA in FEP compared to HC and that GABA levels will positively correlate with FC in HC, but that this relationship will be altered in FEP.

## Methods

### Participants

A total of 177 (HC = 110, FEP = 67) participants were included in this study. Participants overlap with Samson et al. (2025), with additional exclusions made based on FC data quality and availability. FEP patients were recruited from outpatient clinics, inpatient units, and the emergency room at the University of Alabama at Birmingham (UAB). HC were recruited from flyers and ads within UAB. Studies were approved by the UAB Institutional Review Board, and written informed consent was obtained before enrollment; patients had to be deemed competent to provide consent, and parental consent was obtained for patients under the age of 18. Patients were considered eligible for participation if they were between 14 and 55 years old, reported symptoms consistent with a psychotic disorder (e.g. hallucinations, delusions, and negative symptoms), and were interested in study participation.

Exclusion criteria were major neurological or medical conditions, history of significant head trauma, substance use disorders (excluding nicotine and cannabis) within 1 month of imaging, more than 5 days of lifetime antipsychotic exposure, pregnancy or breastfeeding, and MRI contraindications. Use of concomitant medications was permitted as clinically indicated. We did not exclude patients based on a pre-specified maximal duration of untreated psychosis before study entry. Consensus diagnoses were made according to DSM-5 criteria by two board-certified psychiatrists from all historical and direct assessment information available. In addition to the previously mentioned exclusion criteria, HC with a personal or family (first-degree relative) history of a psychiatric illness were also excluded.

The Brief Psychiatric Rating Scale (BPRS) was used to assess psychosis symptom severity in patients (Overall, 1962), where positive subscale, negative subscale, and total scores were used in exploratory analyses. The Repeatable Battery for the Assessment of Neuropsychological Status (RBANS) was used to characterize cognitive function, assessing the domains of immediate memory, visuospatial/constructional, language, attention, and delayed memory in both FEP and HC (Randolph, Tierney, Mohr, & Chase, 1998).

### Data acquisition

**Figure 1.**
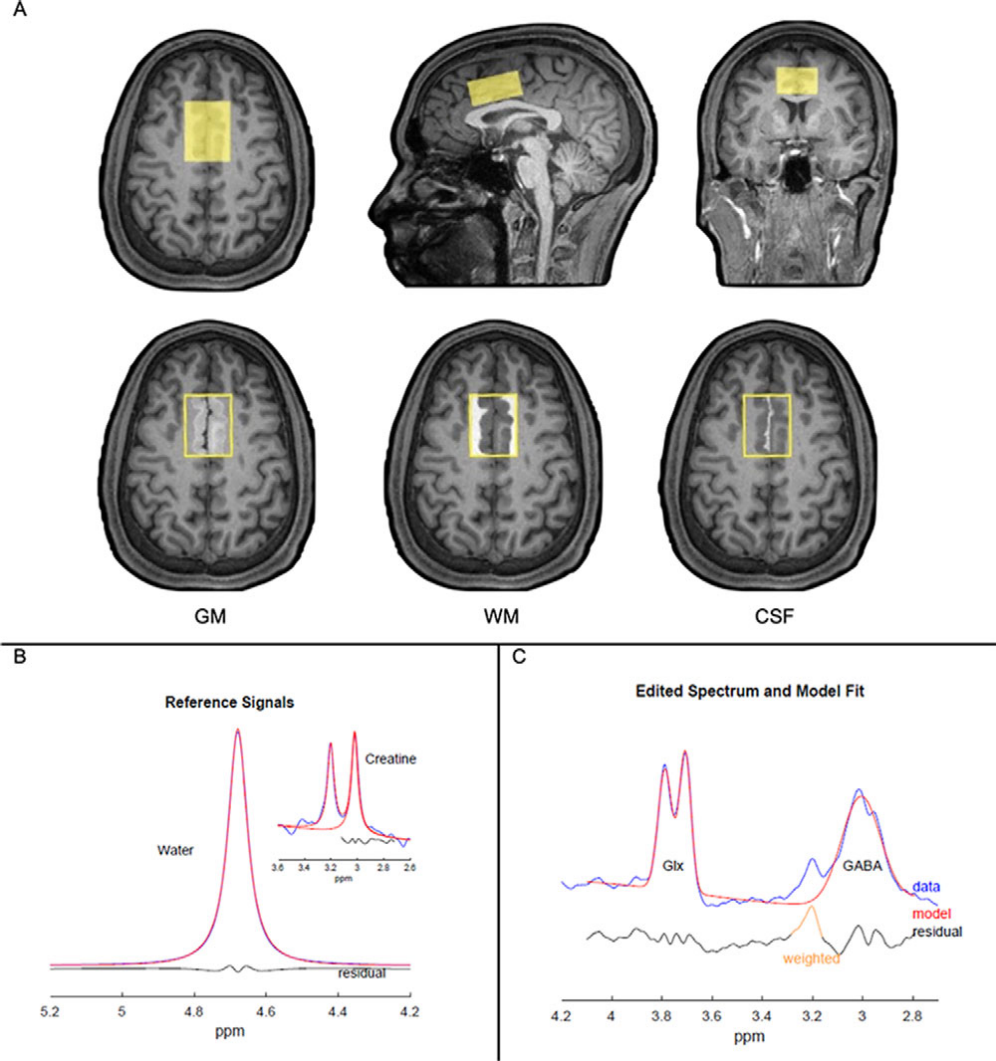
(a) Example voxel placement for the assessment of GABA in the dorsal anterior cingulate cortex (dACC) in one subject. Abbreviations: GM, gray matter; WM, white matter; CSF, cerebrospinal fluid. (b) Reference signals show signal amplitudes for water and creatine, where water signals were used for the estimation of GABA levels. (c) Example of acquired spectrum in one subject, where the electromagnetic signals of specific molecules are used to quantify the concentration of neurometabolites. Blue line shows the collected spectra; red line is a model fit; and black is residual. Peaks at approximately 3.8 parts-per-million (ppm) and 3 ppm indicates measures of Glx (combined glutamate + glutamine) and GABA, respectively. Figure 1. long description.

Imaging was performed on a 3T Siemens MAGNETOM Prisma MRI scanner equipped with a 20-channel head coil. A high-resolution structural T1-weighted scan (MPRAGE: TR = 2400 ms; TE = 2.22 ms; inversion time = 1,000 ms; flip angle = 8°; GRAPPA factor = 2; voxel size = 0.8 mm^3^) was acquired for anatomical reference and to guide voxel placement. MRS data were collected from a voxel in the dACC (voxel size: 30 × 40 × 20 mm^3^, Figure 1a). Outer volume suppression bands were placed close to the edge of the voxel above and below the voxel to reduce unwanted fat signal.

After automated and manual shimming to optimize field homogeneity and water suppression, spectra were acquired using a MEGA-PRESS sequence (TR/TE = 1500/68 ms; with macromolecule suppression; flip angle 90°; 44 Hz bandwidth full width at half maximum editing pulses applied at 1.9 [ON] and 1.5 ppm [OFF]; 256 averages [128 ON and 128 OFF, interleaved]). We also acquired 16 averages of unsuppressed water scans from the same voxel for reference (Figure 1b). Data were saved as single shot spectra (Figure 1c).

### Data preprocessing

#### Magnetic resonance spectroscopy data

MRS data processing methods are detailed in Samson et al. (2025) and summarized here. We performed analyses in MATLAB using Gannet Toolbox. The function GannetLoad was used to import time-domain data and process it into a frequency-domain GABA-edited spectrum. We then used GannetFit to integrate the edited GABA peak at 3 PPM and produce GABA concentration estimates. Through nonlinear least-squares fitting of the spectra, GannetFit estimates the area under the edited GABA signal at 3 PPM and the unsuppressed water signal from the same volume. Quantitative concentrations were calculated as the integral ratio between the GABA area and the water area (Edden et al., 2014). We report GABA concentration relative to water with CSF and tissue correction. We used a complete tissue correction method introduced by Edden et al., dividing the concentration value by the sum of the GM and WM tissue fractions based on the brain segmentation of the ROI in the ACC (Edden et al., 2014; Harris, Puts, & Edden, 2015). The functions GannetCoRegister and GannetSegment were used for brain registration and segmentation, respectively.

For quality control, spectra were checked for presence of residual water, lipid contamination, and spurious echoes. Spectra were excluded if (1) the spectral fit error was greater than 20%, (2) the signal-to-noise ratio (SNR) of the metabolites was less than 3 standard deviations (SDs) below the mean SNR of the HC group (at baseline), or (3) the full width at half maximum (FWHM) of the GABA peak was greater than 3 SDs of the mean FWHM of the HC group.

#### Resting state fMRI data

Functional data acquisition and preprocessing followed similar steps as detailed by Maximo et al. (2021), briefly summarized here. To allow for signal equilibration, the first 10 volumes of each fMRI run were discarded. Spin echo field maps in FSL’s topup were used to correct susceptibility artifacts (Jenkinson et al., 2012). Then, the corrected fMRI runs were concatenated to produce a single 4D image of 430 total volumes. Data were then preprocessed and analyzed using the CONN toolbox (version 21a, https://web.conn-toolbox.org/). Functional images were slice-timing and motion-corrected using rigid-body realignment, then co-registered to the structural image, normalized to Montreal Neurological Institute (MNI) space, low-bandpass filtered (0.008 < *f* < 0.08), and spatially smoothed with a 4-mm FWHM Gaussian kernel.

Framewise displacement (FD) and percentage of censored data were then calculated; participants who had 50% or more data censored were excluded from the study. The Artifact Detection Tools toolbox (NeuroImaging Tools and Resources Collaboratory, https://www.nitrc.org/projects/artifact_detect/) was used to detect and censor motion outliers; participants with composite volume-to-volume motion greater than 0.5 mm or intensity greater than 3 SD from the mean were excluded. Six motion parameters derived from rigid-body realignment and their derivatives, along with the first five component time series derived from CSF and white matter masks from aCompCor and corresponding derivatives, were regressed out from the signal.

### Statistical analyses

Demographic data were compared between groups using a series of independent-sample t-tests and chi-square tests. To compare GABA levels between groups, we performed a one-way ANCOVA controlling for age and sex using the Statistical Package for Social Sciences (SPSS, IBM Corp. Released 2022. IBM SPSS Statistics for macOS, Version 29.0. Armonk, NY: IBM Corp).

To define resting state FC, the following seed regions within the CONN toolbox were used for each network: posterior cingulate cortex (default mode network, DMN), ACC (salience network, SN), right intraparietal sulcus (dorsal attention network, DAN), and right posterior parietal lobule (executive control network, ECN). For FC analyses, residual time series from each ROI were extracted and correlated with every other voxel in the brain, thus creating individual whole-brain z-transformed correlation (positive FC) and anticorrelation (negative FC) maps (Supplementary Figure S1). Then, to restrict FC–GABA analyses to each functional network, masks were applied based on positive and negative FC maps for each network. Masks were created by Maximo et al. (2024) with an overlapping sample of participants by thresholding average correlation maps for each group at *t* = 10 for positive FC, *t* = −20 for negative FC, and a cluster size of 100 voxels and then creating average masks using both groups combined (HC + FEP) for each network, where threshold values were chosen to maximize the field of view for each network without overlapping with white matter or ventricles (Maximo et al., 2024). Group analyses were performed in CONN, restricted to each network mask, using small volume correction (*p* < 0.01 defined by α = 0.05/4 networks of interest), and treating age, sex, and FD as covariates.

To examine GABA’s role in FC, dACC GABA values were used in regression analyses to generate average GABA–FC maps for each subject for each group, for positive and negative FC network masks, while controlling for age, sex, and FD. These maps were entered into 2nd-level analyses to test for group interaction effects in order to find clusters where GABA–FC relationships significantly differ between HC and FEP (*p* < 0.01).

Lastly, exploratory analyses were performed to examine how dACC GABA and FC may relate to symptom severity and cognition. Partial correlations were run to correlate GABA levels with BPRS (positive, negative, and total) scores in patients, as well as with RBANS (subscale and total) scores across groups, while controlling for age and sex. Further, FC z-scores were extracted from clusters of significant GABA–FC association group interactions and correlated with BPRS (in FEP only) and RBANS subscales and total scores (across groups). Cluster FC z-scores analyses were run as bivariate correlations.

## Results

### Demographics and clinical data

We recruited 121 HC and 96 FEP patients for this study. After excluding per quality control methods listed in the data preprocessing section (22 subjects with problematic or incomplete MRS data, 15 with problematic or incomplete fMRI data, and 3 subjects who could not tolerate scanning), our sample included 110 HC and 67 FEP patients. Results from independent-sample *t*- and chi-square tests for demographic data between groups are summarized in Table 1. Groups did not differ in GABA fit error, FWHM, and white matter fraction. FEP patients had significantly higher PPD than HC; HC had higher GABA SNR and gray matter fraction than FEP patients (Table 1).

**Table 1.** Demographics, clinical measures, and data quality^a^ Table 1. long description.

	HC (*n* = 110)	FEP (*n* = 67)	*p*-Value
*Demographic measures*			
Gender, *n* (M/F/O)	59/51/0	35/30/2	0.190
Age, years	23.39 (4.72)	23.82 (5.85)	0.612
Socioeconomic status^b^	4.81 (3.90)	5.94 (5.62)	0.162
Smoking status (no. of packs per day)	0.0056 (0.05)	0.208 (0.37)	<0.001***
*Clinical measures*			
Diagnosis (*n*)			
Schizophrenia	–	31	
Schizoaffective disorder	–	13	
Schizophreniform disorder	–	2	
Bipolar with psychotic features	–	4	
MDD with psychosis	–	1	
Brief psychotic disorder	–	4	
Psychosis not otherwise specified	–	12	
BPRS			
Positive	–	10.57 (3.37)	
Negative	–	5.01 (2.80)	
Total	–	48.16 (10.50)	
RBANS			
Immediate memory	103.41 (14.32)	84.70 (18.77)	<0.001***
Visuospatial	83.95 (13.63)	76.40 (17.55)	0.005**
Language	96.46 (15.83)	84.97 (17.16)	<0.001***
Attention span	103.38 (14.28)	83.78 (17.07)	<0.001***
Delayed memory	91.28 (10.02)	77.35 (16.72)	<0.001***
Total index	93.74 (10.91)	76.32 (15.95)	<0.001***
*Scan quality measures*			
Magnetic resonance spectroscopy			
GABA fit error (%)	4.10 (1.21)	4.34 (1.40)	0.239
GABA FWHM	18.80 (1.51)	18.93 (1.39)	0.563
GABA SNR	29.83 (6.58)	27.25 (5.99)	0.010*
Gray matter fraction (%)	0.533 (0.041)	0.510 (0.036)	<0.001***
White matter fraction (%)	0.374 (0.047)	0.384 (0.045)	0.135
Functional connectivity			
Mean motion (in mm)	0.166 (0.073)	0.184 (0.088)	0.163
% of volumes after scrubbing	93.75 (6.36)	91.40 (8.37)	0.051

a Mean (standard deviation) unless indicated otherwise. *P*-values indicate significance as determined by χ^2^ and independent samples *t*-tests for differences between groups.

b Ranks determined from Diagnostic Interview for Genetic Studies (1–18 scale), where a lower numerical value indicates higher rank, which corresponds to higher socioeconomic status.

(*) indicates significance at *p* < 0.05.

(**) indicates significance at *p* < 0.01.

(***) indicates significance at *p* < 0.001.

Abbreviations: M, male; F, female; O, other (transgender or unspecified); HC, healthy controls; FEP, first-episode psychosis patients; MDD, major depressive disorder; BPRS, Brief Psychiatric Rating Scale; GABA, gamma-aminobutyric acid; RBANS, Repeatable Battery for the Assessment of Neuropsychological Status; FWHM, full width at half maximum; SNR, signal-to-noise ratio; mm, millimeters.

### GABA in HC versus FEP

The one-way ANCOVA found a significant group difference in dACC GABA levels between HC (*M* = 0.595, *SE* = 0.011) and FEP (*M* = 0.558, *SE* = 0.014) after controlling for age and sex (F_1, 173_ = 4.799, *p* = 0.030).

### Network connectivity

**Figure 2.**
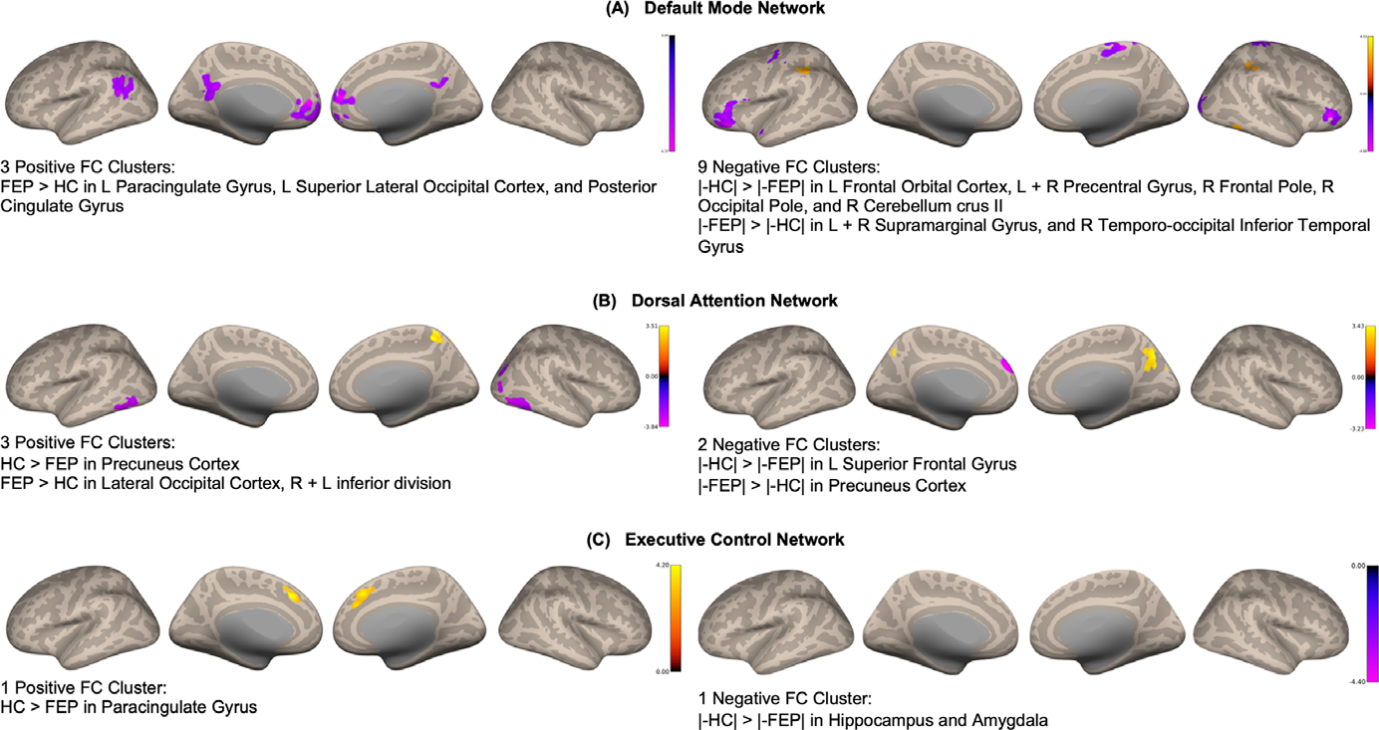
Inflated brain renderings showing clusters where positive or negative FC significantly differed between HC and FEP within the (a) default mode, (b) dorsal attention, and (c) executive control networks. Color bars indicate difference in FC, where warm (red to yellow) clusters indicate where FC is higher in HC compared to FEP, and cool (blue to purple) clusters indicate where FC is higher in FEP compared to HC. Abbreviations: FC, functional connectivity; HC, healthy controls; FEP, first-episode psychosis patients; L, left; R, right. Figure 2. long description.

*Positive FC Masks.* When comparing correlational (positive FC) network maps between groups (Supplementary Figure S1), HC had significantly higher connectivity in the precuneus cortex within the DAN and in the paracingulate gyrus within the ECN. FEP had higher connectivity in the left paracingulate gyrus, left superior division of the lateral occipital cortex, and posterior division of the cingulate gyrus in the DMN, as well as in the bilateral inferior divisions of the lateral occipital cortex in the DAN (Figure 2).

*Negative FC Masks.* For anticorrelation (negative FC) network maps (Supplementary Figure S1), FC was more negative in HC in the left frontal orbital cortex, left and right precentral gyrus, right frontal pole, right occipital pole, and right cerebellum crus 2 in the DMN; in the left superior frontal gyrus in the DAN; and in the hippocampus within the ECN. FEP had more negative FC within the left and right anterior divisions of the supramarginal gyrus, in the right temporo-occipital part of the inferior temporal gyrus in the DMN, and in the precuneus cortex in the DAN (Figure 2).

### GABA and FC associations

**Figure 3.**
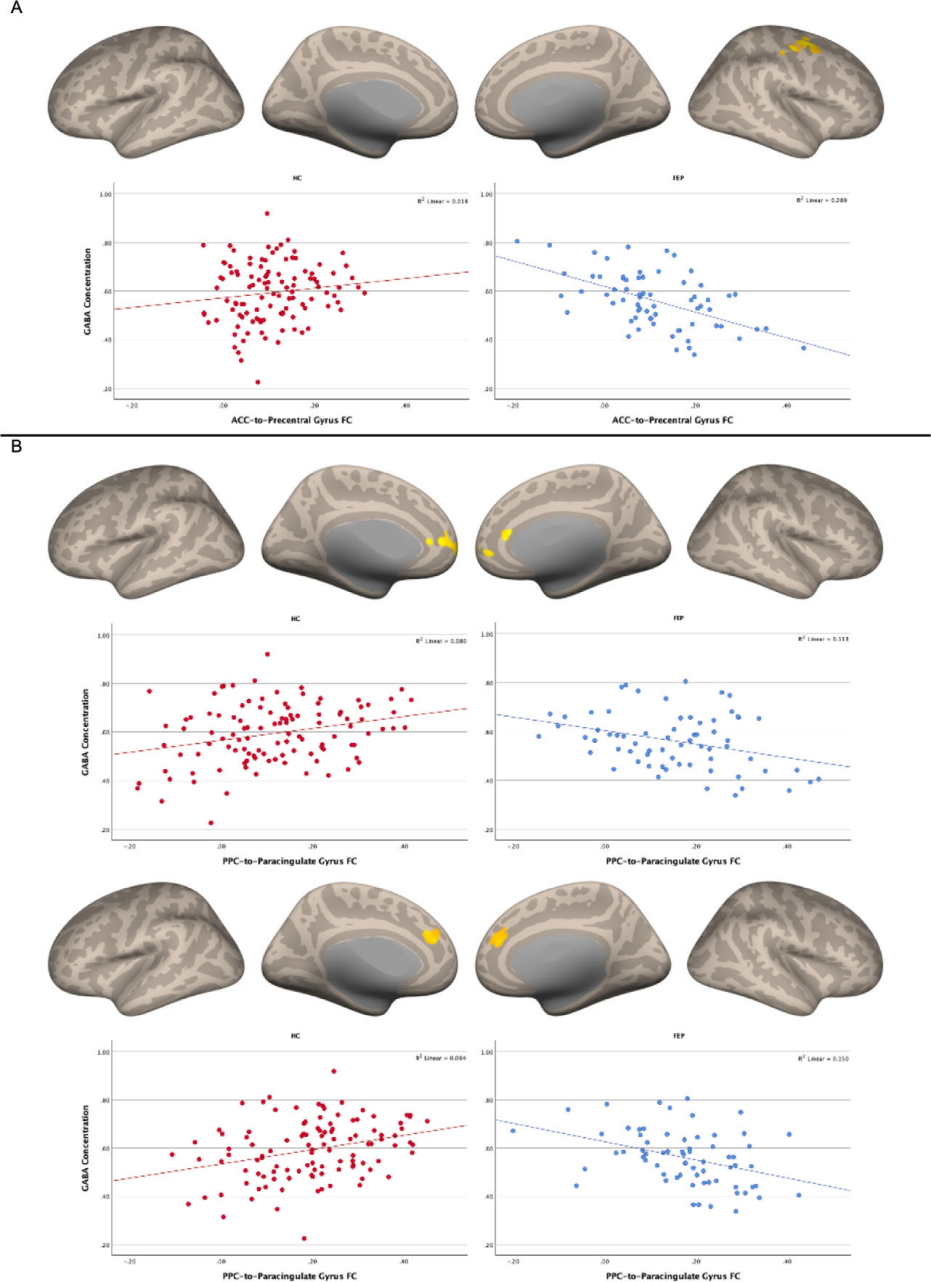
The relationship between FC and GABA was significantly different between HC (left, in red) and FEP (right, in blue), where seed-to-region connectivity from (a) the ACC to one cluster in the right precentral gyrus, and (b) the PPC to two clusters in the bilateral paracingulate gyri positively correlated with GABA in HC, whereas FC and GABA were negatively correlated in FEP. Abbreviations: GABA, gamma-aminobutyric acid; FC, functional connectivity; HC, healthy controls; FEP, first episode psychosis patients; ACC, anterior cingulate cortex; PPC, posterior parietal cortex. Figure 3. long description.

When examining whether GABA concentration correlated with positive or negative FC differently between groups, three significant clusters arose (Table 2 and Figure 3). Within regions associated with the SN, group interaction effects were found in the right precentral, middle frontal, postcentral, and superior frontal gyri, where GABA positively correlated with ACC-to-region FC in HC but negatively correlated in FEP. Similarly, for regions associated with both the ECN and SN, group interactions were found in the bilateral paracingulate gyrus, cingulate gyrus, and left superior frontal gyrus, where GABA positively correlated with right posterior parietal lobule FC in HC, whereas this relationship was negative in FEP (Table 2 and Figure 3).

**Table 2.** Clusters of significant GABA–FC interactions Table 2. long description.

Analysis	Network (seed), region location	MNI coordinates			Cluster size, voxels
		*x*	*y*	*z*	
Positive FC (HC > FEP)	Salience Network (ACC)				
	R precentral gyrus, middle frontal gyrus, postcentral gyrus, superior frontal gyrus	+30	−08	+46	230
	Executive Control Network (PPC)				
	L paracingulate gyrus, R paracingulate gyrus, anterior cingulate gyrus, L frontal pole, R frontal pole	−18	+40	+04	228
	R paracingulate gyrus, L paracingulate gyrus, L superior frontal gyrus	−12	+34	+30	163
Negative FC	N/A	N/A	N/A	N/A	N/A

Abbreviations: GABA, gamma-aminobutyric acid; FC, functional connectivity; MNI, Montreal Neurological Institute; HC, healthy control; FEP, first-episode psychosis patients; ACC, anterior cingulate cortex; PPC, posterior parietal cortex; R, right; L, left.

### Exploratory analyses

After controlling for age and sex, partial correlations found that dACC GABA negatively correlated with BPRS negative scores for FEP (*r*(63) = −0.346, *p* = 0.005) and dACC GABA positively correlated with RBANS Attention subscale scores across groups (*r*(163) = 0.176, *p* = 0.024). Bivariate correlations found that FC z-scores in one ECN cluster (whose peak location was in the right paracingulate gyrus, abbreviated as cluster ECN 2 in Supplementary Table S2) positively correlated with Visuospatial/Constructional (*r* = 0.188, *p* = 0.015), Attention (*r* = 0.170, *p* = 0.028), and Total (*r* = 0.190, *p* = 0.014) RBANS scores. After correction for multiple comparisons, only the correlation between dACC GABA and BPRS negative scores remained significant; all other correlations did not survive multiple comparison correction. Partial and bivariate correlation and significance values are listed in Supplementary Table S2.

## Discussion

In this study, we investigated GABAergic differences and how they relate to higher-order functional network connectivity in a large sample of antipsychotic medication-naïve patients with first-episode psychosis (FEP), compared to HC. We report significant group differences in dACC GABA concentrations, where FEP had lower GABA levels compared to HC. We also found several clusters in the ECN, DAN, and DMN where positive and negative FC significantly differed between HC and FEP. Importantly, we found significant GABA-FC group interactions in two clusters associated with the ECN and one cluster associated with the SN, where GABA and FC were positively correlated in HC but negatively correlated in FEP.

As GABA’s principal role in the brain is inhibition, it is perhaps unsurprising that in HC, GABA concentration has a positive relationship with FC in the SN, a network primarily involved in detecting, evaluating, and filtering salient sensory data. Similarly, GABA levels positively correlate with FC in the ECN, which is responsible for cognitive control, working memory, and problem solving. For HC, higher ACC GABA levels were associated with higher network FC, so better inhibitory function here is associated with healthy cognitive function. However, we found the opposite in patients, where GABA levels negatively correlated with network FC. Thus, in the context of disrupted GABA neurotransmission in FEP, we observed functional correlates consistent with excitatory-inhibitory (E/I) imbalance. Typically, GABAergic signaling is associated with inhibitory stabilization, allowing for better spatiotemporal coordination throughout excitatory neurons in cortical circuits (Kirmse & Zhang, 2022). Aberrancies in the E/I balance have been implicated in schizophrenia, where genetic vulnerability along with environmental risk factors affecting glutamatergic and GABAergic signaling can lead to abnormal synaptic refinement, preventing normal neurodevelopment (Howes & Shatalina, 2022). Our findings add to the growing literature of inter-network dysregulation and systems-level disruption among the salience, central executive, and default mode networks contributing to psychosis symptoms (Supekar et al., 2019) as described by the triple network model of aberrant saliency mapping and cognitive dysfunction in psychopathology (Menon, 2019; Menon, 2011).

Notably, our exploratory analyses did not find significant correlations between GABA and cognition as measured by RBANS subscales and total scores. Further, based on BPRS scores in FEP, GABA significantly correlated with negative symptoms, where lower GABA levels were associated with more negative symptoms. In conjunction with findings reporting GABA differences between patients grouped by treatment response, where patients who did not respond well to treatment had lower GABA levels at baseline (Bojesen et al., 2020; Samson et al., 2025), these data suggest that GABA levels in the ACC may be a useful predictor of symptomatology and response to treatment.

We, in an overlapping sample of patients (Samson et al., 2025), and others (Bojesen et al., 2020; Bojesen et al., 2024) have reported lower ACC GABA levels in medication-naïve FEP. Likewise, several studies have reported altered connectivity in higher-order functional brain networks in psychosis (Maximo et al., 2024; Xiang et al., 2019; Zhang et al., 2022). Our findings support a growing body of evidence of GABA’s significant and differential role in FC in vivo in psychosis spectrum disorders. Importantly, GABA works in concert with glutamate, so we highlight investigating the role of E/I balance in FC. Maximo et al. found altered associations between dACC glutamate + glutamine (Glx) and FC, where Glx was associated with positive and negative FC in the DMN, DAN, and ECN for HC, but these relationships were altered or absent in antipsychotic medication-naïve FEP (Maximo et al., 2024). In studies comparing medicated FEP to HC, Overbeek et al. also found that relationships between ACC glutamate, GABA, and FC differed between groups (Overbeek et al., 2021) and that ACC glutamate and GABA differentially correlated with BOLD response in regions of the ECN and DMN (Overbeek et al., 2019). Ultimately, both glutamate and GABA levels relate to FC, and so psychosis can be characterized as a product of both E/I imbalance and disordered brain network organization (Liu et al., 2021; O’Neill, Mechelli, & Bhattacharyya, 2019).

Overall, this calls for further investigation of the relationship between neurometabolic function and FC within and between these networks, and how these mechanisms differ between FEP and HC. Our findings should be considered within the context of several strengths and limitations. To our knowledge, this is one of the largest studies examining the relationship between GABA and FC in antipsychotic medication-naïve FEP. Strengths of this study include mitigation of the potential confounds of antipsychotic medication effects and illness chronicity; a possible weakness of this early psychosis cohort is the heterogeneity of diagnoses, as variability between diagnoses may influence observed effects. Another strength of our study is that we utilize a MEGA-PRESS sequence for GABA measurement, which includes macromolecule suppression by editing pulses, thus eliminating macromolecule contamination and increasing clarity of results and statistical power (Edden, Puts, & Barker, 2012; Mikkelsen, Harris, Edden, & Puts, 2018). Further, we did not exclude or match participants on the basis of nicotine or cannabis use. Research has established that nicotine use is consistently more prevalent in patients with psychosis spectrum disorders (de Leon & Diaz, 2005; Gurillo, Jauhar, Murray, & MacCabe, 2015), and cannabis is a major risk factor for the development of psychosis (Arseneault, Cannon, Witton, & Murray, 2004; Casadio, Fernandes, Murray, & Di Forti, 2011), so inclusion of these patients is clinically relevant, and exclusion would inadvertently bias our sample and limit the generalizability of our findings. Another limitation is the higher exclusion rate of patients’ imaging data, especially neurometabolite spectra, as data quality can be significantly impacted by movement, and more patients may differ in MRI tolerance. Additionally, MRS voxels were placed in the native space for each participant, whereas FC analyses were done in the MNI space, where the salience network typically included most of the ACC. Further investigation would benefit from analysis of the degree of overlap between voxel placement within FC network masks, in order to better interpret the relationship between voxel-level neurochemistry and network FC.

Future studies should consider heterogeneity within patient populations, acknowledging the potential effects of treatment (type, duration, and dosage) and illness chronicity. Our data found significant interactions between group and GABA on FC in patients at baseline, so more extensive research is merited regarding how these measures may change over time, correlate with antipsychotic treatment, predict treatment response, and differ between presentations of psychosis. Additionally, the E/I balance should be investigated, taking into account glutamate levels, considering glutamate-to-GABA as a ratio, and examining how neurotransmission of these metabolites in different regions may have varying effects on cognitive and behavioral function. Bojesen et al. (2026) have found an aberrant relationship between dopamine synthesis GABA in antipsychotic-naïve FEP, and so further analysis including combined neurotransmitter disturbances is merited (Bojesen et al., 2026). Large sample sizes with similarly stringent patient inclusion criteria and more powerful MRI technology (e.g. 7 T) would further shed light on the pathophysiology of psychosis spectrum disorders and aid in our understanding of these mechanisms and the development of treatments that specifically target these abnormalities which can be consistently found in FEP.

## Conclusion

In summary, we found that dACC GABA, higher-order functional network FC, and the relationships between GABA and FC are significantly altered in antipsychotic medication-naïve patients at their first psychotic episode. Our data add to the literature demonstrating that FC should be considered when characterizing these brain networks and that the E/I balance plays a significant role in network connectivity. Investigation of GABAergic dysfunction and aberrancies in FC is critical to explaining the symptomatology and development of psychosis spectrum disorders and will be important for the development of targeted, effective treatments.

## Supporting information

10.1017/S0033291726104358.sm001 Samson et al. supplementary material Samson et al. supplementary material

## Supplementary material

The supplementary material for this article can be found at http://doi.org/10.1017/S0033291726104358.

## Table 1. Long description

The table is organized into three main sections: demographic measures, clinical measures, and scan quality measures. Each section lists variables in the leftmost column, with data for healthy controls (n equals 110) in the second column, first-episode psychosis patients (n equals 67) in the third, and p-values in the fourth. Demographic measures include gender (healthy controls: 59 male, 51 female, 0 other; first-episode psychosis: 35 male, 30 female, 2 other; p equals 0.190), age (healthy controls: 23.39 years, standard deviation 4.72; first-episode psychosis: 23.82, standard deviation 5.85; p equals 0.612), socioeconomic status (healthy controls: 4.81, standard deviation 3.90; first-episode psychosis: 5.94, standard deviation 5.62; p equals 0.162), and smoking status (healthy controls: 0.0056 packs per day, standard deviation 0.05; first-episode psychosis: 0.208, standard deviation 0.37; p less than 0.001, triple asterisk). Clinical measures list diagnoses only for first-episode psychosis: schizophrenia (31), schizoaffective disorder (13), schizophreniform disorder (2), bipolar with psychotic features (4), major depressive disorder with psychosis (1), brief psychotic disorder (4), psychosis not otherwise specified (12). BPRS scores for first-episode psychosis are positive: 10.57 (standard deviation 3.37), negative: 5.01 (standard deviation 2.80), total: 48.16 (standard deviation 10.50). RBANS scores show healthy controls outperform first-episode psychosis patients in immediate memory (103.41 vs 84.70, p less than 0.001, triple asterisk), visuospatial (83.95 vs 76.40, p equals 0.005, double asterisk), language (96.46 vs 84.97, p less than 0.001, triple asterisk), attention span (103.38 vs 83.78, p less than 0.001, triple asterisk), delayed memory (91.28 vs 77.35, p less than 0.001, triple asterisk), and total index (93.74 vs 76.32, p less than 0.001, triple asterisk). Scan quality measures include GABA fit error (healthy controls: 4.10 percent, standard deviation 1.21; first-episode psychosis: 4.34, standard deviation 1.40; p equals 0.239), GABA FWHM (18.80 vs 18.93; p equals 0.563), GABA SNR (29.83 vs 27.25; p equals 0.010, single asterisk), gray matter fraction (0.533 vs 0.510; p less than 0.001, triple asterisk), white matter fraction (0.374 vs 0.384; p equals 0.135), mean motion in millimeters (0.166 vs 0.184; p equals 0.163), and percent of volumes after scrubbing (93.75 vs 91.40; p equals 0.051). Significant group differences are marked with asterisks according to p-value thresholds. Abbreviations are defined in the table footnote.

Navigate back to Table 1..

## Table 2. Long description

The table is organized by analysis type, with ‘Positive F C (H C greater than F E P)' first. Under the Salience Network (A C C), the right precentral gyrus, middle frontal gyrus, postcentral gyrus, and superior frontal gyrus have M N I coordinates x plus 30, y minus 08, z plus 46, cluster size 230 voxels. For the Executive Control Network (P P C), the left and right paracingulate gyri, anterior cingulate gyrus, left and right frontal poles have coordinates x minus 18, y plus 40, z plus 04, cluster size 228 voxels. Another cluster in the right and left paracingulate gyri and left superior frontal gyrus has coordinates x minus 12, y plus 34, z plus 30, cluster size 163 voxels. The ‘Negative F C’ row lists all entries as N slash A. Abbreviations are defined below the table.

Navigate back to Table 2..

## Figure 1. Long description

There are three MRI panels arranged horizontally. The leftmost panel shows an axial brain slice with a yellow rectangular voxel centered in the dorsal anterior cingulate cortex. The middle panel presents a sagittal view, again highlighting the same region with a yellow voxel, demonstrating its anterior-posterior and superior-inferior boundaries. The rightmost panel displays a coronal slice, with the yellow voxel positioned symmetrically above the corpus callosum. The underlying brain tissue is visible in shades of gray, with clear delineation of gray matter, white matter, and cerebrospinal fluid. The yellow voxel is consistently placed in all three views, confirming its anatomical localization.

Navigate back to Figure 1..

## Figure 2. Long description

From left to right, eight cortical surface renderings display the left and right hemispheres in lateral and medial views. Each panel is labeled as part of the default mode network. Purple to blue clusters indicate regions where functional connectivity is higher in first-episode psychosis patients compared to healthy controls, while yellow to red clusters indicate regions where functional connectivity is higher in healthy controls. The clusters are distributed across the medial prefrontal, posterior cingulate, and lateral parietal cortices. A vertical color bar at the far right ranges from purple (lowest difference) through yellow (highest difference), mapping the magnitude and direction of group differences in functional connectivity.

Navigate back to Figure 2..

## Figure 3. Long description

From left to right, three horizontal panels display dorsal views of the brain. The first two panels show anatomical outlines without overlays. The third panel, at the far right, contains a yellow cluster in the right precentral gyrus, marking a region of significant functional connectivity difference. The cluster is positioned laterally and slightly anterior on the right hemisphere. No other regions are highlighted in these panels. The background is neutral, and no additional text or legends are present within the panels.

Navigate back to Figure 3..
