# Supplementary material for: Network functional connectivity and anterior cingulate cortex gamma-aminobutyric acid in antipsychotic medication-naïve first-episode psychosis patients
Source: Psychol Med. 2026 Jun 9;56:e187. doi: 10.1017/S0033291726104358 (PMC13247797; doi:10.1017/S0033291726104358)
Supplement: Samson et al. supplementary material [file S0033291726104358sup001.docx]

**SUPPLEMENTARY MATERIALS**

**Figure S1.** Positive and negative functional connectivity per network for each group

| 1. **Default Mode Network**  \| **HC** \| **FEP** \| \| --- \| --- \| | |
| --- | --- | --- | --- |
| **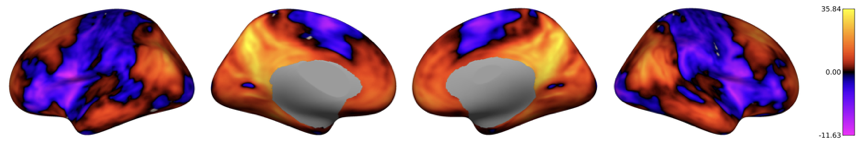** | **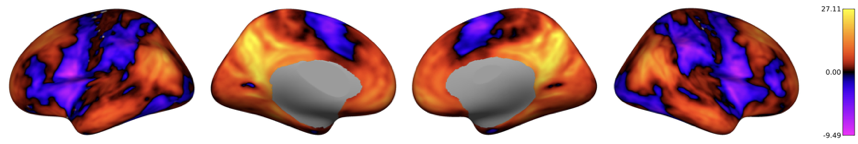** |
| 1. **Dorsal Attention Network** | |
| **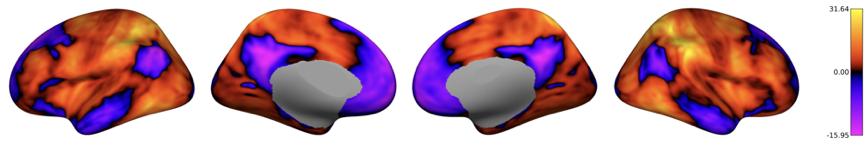** | **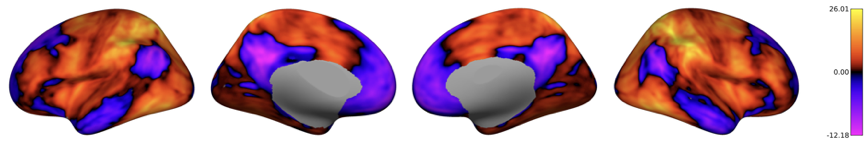** |
| 1. **Executive Control Network** | |
| **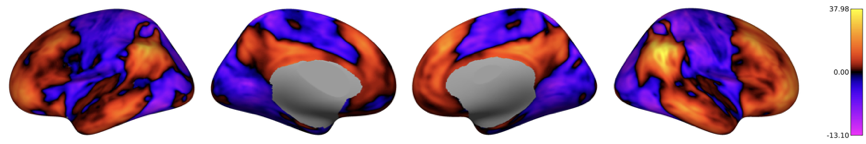** | **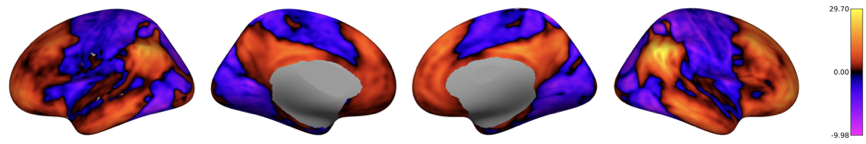** |
| 1. **Salience Network** | |
| **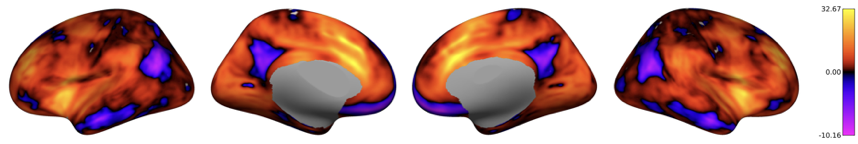** | **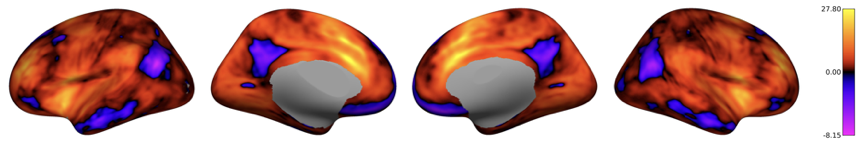** |

**Fig. S1.** Inflated brain renderings showing maps of positive and negative FC in the (A) default mode, (B) dorsal attention, (C) executive control, and (D) salience networks, for HC (left) and FEP (right). Color bars indicate seed-to-voxel FC, where warm (red to yellow) indicates positive FC, and cool (blue to purple) indicates negative FC. Abbreviations: FC, functional connectivity; HC, healthy controls; FEP, first-episode psychosis patients.

**Table S2.** dACC GABA and Cluster FC Correlations with BPRS and RBANS

|  |  |  | **FC Cluster** | | |
| --- | --- | --- | --- | --- | --- |
|  |  | **dACC GABA** | **SN** | **ECN 1** | **ECN 2** |
| **BPRS** (n = 67 FEP) |  |  |  |  |  |
| **Positive** | Correlation  *p-value* | -.173  .167 | .129  .297 | .076  .541 | .069  .578 |
| **Negative** | Correlation  *p-value* | -.346**  .005 | -.096  .439 | .113  .362 | .119  .339 |
| **Total** | Correlation  *p-value* | -.166  .186 | -.037  .766 | .081  .517 | .099  .425 |
|  |  |  |  |  |  |
| **RBANS** (n = 107 HC, 60 FEP) |  |  |  |  |  |
| **Immediate Memory** | Correlation  *p-value* | -.024  .757 | -.055  .479 | .013  .867 | .152  .050 |
| **Visuospatial/Constructional** | Correlation  *p-value* | -.051  .512 | -.008  .919 | .029  .709 | .188*  .015 |
| **Language** | Correlation  *p-value* | .024  .763 | -.007  .924 | -.090  .247 | .064  .408 |
| **Attention** | Correlation  *p-value* | .176*  .024 | -.040  .610 | .014  .855 | .170*  .028 |
| **Delayed Memory** | Correlation  *p-value* | .096  .222 | -.143  .065 | -.027  .729 | .111  .153 |
| **Total** | Correlation  *p-value* | .061  .433 | -.057  .465 | -.011  .890 | .190*  .014 |

Correlation and significance (2-tailed) values for baseline dACC GABA levels and FC cluster z-scores with BPRS (patients only) and RBANS scores (across groups). “ECN 1” refers to the cluster whose peak location was in the left paracingulate gyrus; “ECN 2” refers to that in the right paracingulate gyrus.

Abbreviations: dACC, dorsal anterior cingulate cortex; GABA, gamma-aminobutyric acid; FC, functional connectivity; BPRS, Brief Psychiatric Rating Scale; RBANS, Repeatable Battery for the Assessment of Neuropsychological Status; SN, salience network; ECN, executive control network

(*) indicates significance at p < .05.

(**) indicates significance at p < .01.
